# Assessing Overall Exercise Recovery Processes Using Carbohydrate and Carbohydrate-Protein Containing Recovery Beverages

**DOI:** 10.3389/fphys.2021.628863

**Published:** 2021-02-04

**Authors:** Isabella Russo, Paul A. Della Gatta, Andrew Garnham, Judi Porter, Louise M. Burke, Ricardo J. S. Costa

**Affiliations:** ^1^Department of Nutrition and Dietetics, Monash University, Notting Hill, VIC, Australia; ^2^Institute for Physical Activity and Nutrition, School of Exercise and Nutrition Sciences, Deakin University, Geelong, VIC, Australia; ^3^Mary MacKillop Institute for Health Research, Australian Catholic University, Melbourne, VIC, Australia

**Keywords:** hydration, muscle glycogen, mTOR, gastrointestinal, immune, inflammation

## Abstract

We compared the impact of two different, but commonly consumed, beverages on integrative markers of exercise recovery following a 2 h high intensity interval exercise (i.e., running 70–80% *V̇*O_2__max_ intervals and interspersed with plyometric jumps). Participants (*n* = 11 males, *n* = 6 females) consumed a chocolate flavored dairy milk beverage (CM: 1.2 g carbohydrate/kg BM and 0.4 g protein/kg BM) or a carbohydrate-electrolyte beverage (CEB: isovolumetric with 0.76 g carbohydrate/kg BM) after exercise, in a randomized-crossover design. The recovery beverages were provided in three equal boluses over a 30 min period commencing 1 h post-exercise. Muscle biopsies were performed at 0 h and 2 h in recovery. Venous blood samples, nude BM and total body water were collected before and at 0, 2, and 4 h recovery. Gastrointestinal symptoms and breath hydrogen (H_2_) were collected before exercise and every 30 min during recovery. The following morning, participants returned for performance assessment. In recovery, breath H_2_ reached clinical relevance of >10 ppm following consumption of both beverages, in adjunct with high incidence of gastrointestinal symptoms (70%), but modest severity. Blood glucose response was greater on CEB vs. CM (*P* < 0.01). Insulin response was greater on CM compared with CEB (*P* < 0.01). *Escherichia coli* lipopolysaccharide stimulated neutrophil function reduced on both beverages (49%). p-GSK-3β/total-GSK-3β was greater on CM compared with CEB (*P* = 0.037); however, neither beverage achieved net muscle glycogen re-storage. Phosphorylation of mTOR was greater on CM than CEB (*P* < 0.001). Fluid retention was lower (*P* = 0.038) on CEB (74.3%) compared with CM (82.1%). Physiological and performance outcomes on the following day did not differ between trials. Interconnected recovery optimization markers appear to respond differently to the nutrient composition of recovery nutrition, albeit subtly and with individual variation. The present findings expand on recovery nutrition strategies to target functionality and patency of the gastrointestinal tract as a prerequisite to assimilation of recovery nutrition, as well as restoration of immunocompetency.

## Introduction

The manipulation of recovery nutrition to promote physiological restoration, adaptation to training and, potentially, performance benefits is an established practice amongst athletes and a key theme in sports nutrition research. Over the past decade, studies have meticulously investigated the optimal nutritional approach for isolated goals, such as, the replacement of energy substrate and body water losses, and repair of damaged tissues (e.g., skeletal muscle), leading to the development of generalized recommendations for each element of exercise recovery (Thomas et al., 2016). Such guidelines target individual aspects of exercise recovery, offering prescriptive values for the intakes of carbohydrate, protein, and water to support muscle and liver glycogen replenishment, skeletal muscle protein synthesis, and rehydration, respectively. To date, however, recovery guidelines have failed to consider the impact of exercise-induced gastrointestinal syndrome (EIGS) on regulation of nutrient availability via gastrointestinal integrity and functional responses and (or) the restoration of immunocompetency in response to immunodepressive exercise (Costa et al., 2017a, 2020a; Peake et al., 2017). Yet, these neglected aspects appear to be associated with nutrient circulatory and cellular bioavailability and subsequently determines the overall recovery nutrition outcomes (Russo et al., 2019).

It is well established that prolonged strenuous exercise reduces muscle glycogen and body water content, and induces skeletal muscle damage. For example, steady state treadmill running for 90 min at 70% *V̇*O_2__max_ in 20–22°C ambient temperature (*T*_amb_) and 54–56% relative humidity (RH) has been reported to deplete muscle glycogen content below 250 mmol/kg dry weight (dw), and induce body mass (BM) loss >2.5% indicative of mild dehydration (Wong and Williams, 2000; Alghannam et al., 2016). Muscle damage, as indicated by biochemical [i.e., increased creatine kinase (CK) and (or) myoglobin] and functional [i.e., reduced isometric maximal voluntary contraction and (or) increased muscle soreness] markers is induced by eccentric plyometric contractions (i.e., 5 × 20 drop jumps) and prolonged intermittent running (i.e., 90 min Loughborough Intermittent Shuttle Test) (Miyama and Nosaka, 2004; Magalhães et al., 2010). From an immune perspective, prolonged strenuous exercise is also known to induce a systemic leukocytosis, and depress several immune cell functional responses (i.e., reduced bacterially stimulated neutrophil degranulation *in vitro*, reduced mitogen or antigen-induced lymphocyte proliferation *in vitro*, and reduced antigen-induced delayed hypersensitivity challenge *in vivo*), the extent of which appears to be proportional to exercise intensity and duration (Walsh et al., 2011b). Impaired immunocompetency, especially neutrophil functional responses, may result in suboptimal clearance of damaged cell debris required for muscle glycogen resynthesis (Burke et al., 2017; Suzuki, 2018), tissue repair and remodeling (Tidball and Villalta, 2010), and clearance of environmental and (or) luminal-derived pathogenic agents (Costa et al., 2017a, 2019; Snipe and Costa, 2018a; Snipe et al., 2018a). Indeed, bacteria and bacterial endotoxins may enter systemic circulation by physical breaks and (or) hyperpermeability in the gastrointestinal epithelium as a result of EIGS, leading to a pronounced systemic inflammatory response and (or) gastrointestinal symptoms (GIS) (Snipe et al., 2017, 2018a; Bennett et al., 2020; Suzuki et al., 2020). The recovery of these exercise-induced physiological disturbances, returning to baseline levels and (or) assisting with adaptations to the exercise stress, is highly dependent on nutrient bioavailable during passive rest. Moreover, recovery outcomes are ultimately regulated by food and fluid choice and ingestion (i.e., nutrient density and water volume), and gastrointestinal functional responses to the intake load.

Circulatory nutrient availability is a key feature in immunocompetence during and after prolonged strenuous exercise. For example, frequent carbohydrate ingestion (i.e., 45 g/h) during exercise has shown favorable effects by attenuating perturbations to bacterial endotoxin and systemic inflammatory profiles (Snipe et al., 2017). While, carbohydrate [i.e., 1.2 g/kg body mass (BM)], with or without protein (i.e., 0.4 g/kgBM) provisions immediately post-exercise has also shown favorable effects in preventing post-exercise reduction in neutrophil functional, albeit *in vitro* (Costa et al., 2009, 2011, 2020a), but not other immune functional responses and (or) status concentration changes (Costa et al., 2011, 2020a). Ultimately these immune restoration outcomes are dependent on the regulation of the gastrointestinal tract in allowing circulatory nutrient availability. As such, EIGS can impair gastrointestinal functional capacity leading to reduced post-exercise nutrient bioavailability (Lang et al., 2006; van Wijck et al., 2013; Costa et al., 2017b, 2020a). Failure to consider the influence of EIGS on nutrient intake, bioavailability and cellular asimilation may have led to suboptimal estimates for acute recovery nutrition guidelines and recommendations targeting glycogen replenishment and tissue repair. Considering the integrative and inter-dependant nature of exercise recovery processes (i.e., gastrointestinal patency, immune restoration, rehydration, muscle glycogen and protein resynthesis), in assessing the impact of food and (or) fluid provisions after exercise on overall recovery, it is imperative to apply an exercise stress model known to perturb the physiological status of these recovery categories. As such, it appears exercise duration up to 2 h, of intermittent high intensity nature, and inclusion of plyometric jumps meets this criteria (Miyama and Nosaka, 2004; Costa et al., 2009, 2011, 2020b; Snipe et al., 2017, 2018a,b; Russo et al., 2019).

Contemporary sports nutrition recognizes the need to personalize and periodise the nutrition support for training and competition, including recovery nutrition, whether it refers to acute (e.g., 4–8 h) or longer-term (e.g., following day or >24 h) recovery processes. The concept of ‘exercise recovery optimization’ provides an additional contribution to the sophistication of our knowledge and practice, by integrating recovery strategies that maximize desired outcomes while minimizing those that cause detrimental outcomes within the complex and interrelated recovery responses to exercise (Russo et al., 2019). It is now well established and generally accepted that the foods and fluids provided immediately after exercise cessation play an important role in subsequent physical performance outcomes when repetitive bouts have a shorter time separation (e.g., several hours), but are less impacting on performance outcomes over longer-periods (e.g., next day and onward) when habitual dietary intake may support the overall recovery processes (McCartney et al., 2018; Wan et al., 2018; Russo et al., 2019). Firstly, it is still unknown how exercise-associated gastrointestinal and immune perturbations impacts recovery nutrition processes and subsequent performance (i.e., the following day), using adequate dietary control experimental models. Secondly, from a practical perspective, daily repetitive exercise bouts of sufficient exertion (e.g., duration and intensity) to induce physiological disturbance that warrants recovery attention is normally associated to training loads, whilst competition is habitually on consecutive days. As such, merging relevant nutritional provisions, recovery process, and timescale seems the most logical translational aspect of professional practice.

Considering the importance of carbohydrate, protein and water provisions, and nutrient bioavailability in supporting exercise recovery processes, flavored dairy milk beverages and carbohydrate electrolyte beverages are the most commonly consumed beverages amongst athletic populations in the post-exercise recovery period, despite their isovolumetric energy and nutritional composition differences (Roy, 2008; Russo et al., 2019). These nutritionally diverse, but intake tolerable beverages, provide an ideal nutritional comparison to assess nutrient availability and overall exercise recovery variables (e.g., carbohydrate-protein vs. carbohydrate). With this in mind, the current study aimed to investigate the impact of a carbohydrate- and protein-containing flavored dairy beverage and a non-nitrogenous carbohydrate electrolyte beverage on overall and integrative markers of acute recovery following an exercise stress known to perturb many aspects of physiological and metabolic homeostasis. The influence of physiological and nutritive factors on assimilation and bioavailability of recovery nutrition was assessed using global markers of recovery optimization including EIGS, immune function response previously confirmed to depress after exercise and response to recovery nutrition (e.g., *in vitro* bacterially-stimulated neutrophil elastase release), muscle glycogen resynthesis, protein synthesis, rehydration, and performance outcomes. It was hypothesized that nutrient malabsorption associated with EIGS would result in gastrointestinal symptoms (GIS), impair total nutrient availability, and sub-optimize acute recovery outcomes. It was also hypothesized that the overall greater nutrient content of the dairy (carbohydrate-protein containing) beverage would result in a greater overall benefit on recovery markers than carbohydrate alone, and result in greater exercise performance the following day.

## Materials and Methods

### Participants

Seventeen (*n* = 11 males, *n* = 6 females) recreationally trained endurance athletes [mean (*SD*): age 33.7 (9.3) years, nude BM 69.1 (10.1) kg, height 173.9 (8.1) cm,% body fat 17.8 (6.6)%, *V̇*O_2__max_ 55.8 (6.9) ml/kg BM/min, weekly training volume 413 (201) min, and modality: endurance running, endurance cycling, ultra-endurance running, HIIT] volunteered to participate in the study and completed the experimental procedures. All participants gave written informed consent. The study protocol received approval from the local ethics committee (Monash University Human Research Ethics Committee: 12799) and conformed with the Helsinki Declaration for Human Research Ethics. The trial was prospectively registered with ANZCTR (reference number 375090). All participants confirmed being free from illness, disease [including gastrointestinal infections, diseases and (or) disorders] and injury. Individuals were excluded if they confirmed having consumed potential dietary modifiers of gastrointestinal integrity, were adhering to gastrointestinal-focused dietary regimes within the previous 3 months, or consumed non-steroidal anti-inflammatory medications, antibiotic and (or) stool altering medications within 1 month before the experimental protocol. An additional 4 participants were recruited but were unable to complete both trials, due to illness/injury (*n* = 1 male, *n* = 1 female) or unexpected unavailability (*n* = 1 male, *n* = 1 female).

### Preliminary Measures

One to three weeks prior to the first experimental trial, baseline measurements for height (Stadiometer, Holtain Limited, Crosswell, Crymych, United Kingdom), BM (Seca 515 MBCA, Seca Group, Hamburg, Germany), body composition (Seca 515 MBCA, Seca Group, Hamburg, Germany) and *V̇*O_2__max_ (Vmax Encore Metabolic Cart, Carefusion, San Diego, CA, United States) were recorded. *V̇*O_2__max_ was estimated by means of a continuous incremental exercise test to volitional exhaustion on a motorized treadmill (Forma Run 500, Technogym, Seattle, WA, United States), as previously reported (Costa et al., 2009). Criteria for attaining *V̇*O_2__max_ included the participant reaching volitional exhaustion [i.e., rating of perceived exertion (RPE) of 19–20 Borg scale], a heart rate (HR) within 10 beats/min of HR_max_, with observation of *V̇*O_2_ plateau is increasing exercise intensity and/or inclusion of RER (≥1.10). To determine running speeds for the exercise trials, the speed at approximately 50 [7.3 (1.0) km/h], 55–60 [8.7 (1.3) km/h], 70–75 [10.8 (1.4) km/h], and 80–85 [12.7 (1.8) km/h]% *V̇*O_2__max_ and 1% gradient was determined and verified from the *V̇*O_2_-work rate relationship.

### Experimental Protocol

**FIGURE 1:**
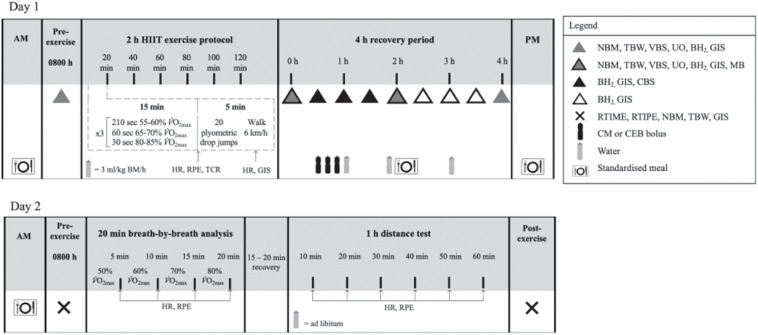
Schematic illustration of the experimental design. NBM, nude body mass; TBW, total body water; VBS, venous blood sampling; UO, urine output and osmolality; BH_2_, breath hydrogen; GIS, gastrointestinal symptoms; HR, heart rate; TCR, thermal comfort rating; RPE, rating of perceived exertion; MB, muscle biopsy; CBS, capillary blood sampling; RTIME, readiness to invest mental effort; RTIPE, readiness to invest physical effort; RER, respiratory exchange ratio; CM, chocolate flavored dairy milk; CEB, carbohydrate-electrolyte beverage; BM, body mass.

To control dietary intake, all foods and fluids were provided throughout the experimental trials, and participants were required to consume a standardized low fermentable carbohydrate (FODMAP) diet during the 24 h before, and throughout the experimental trials. Meals were designed in accordance with current nutrition guidelines for endurance athletes, and calculated to provide <2 g FODMAP per meal using a FODMAP specific database (Monash University, FoodWorks Professional 7, Xyris, Brisbane, Australia) (Thomas et al., 2016; Gaskell et al., 2020b). Compliance was assessed using a food and fluid diary [overall mean (SD): energy 10.1 (3.0) MJ/day, protein 98 (30) g/day, fat 57 (36) g/day, carbohydrate 353 (87) g/day, fiber 44 (11) g/day, and water 2,333 (1,358) ml/day]. Participants were asked to avoid alcohol and strenuous exercise during the 48 h before each experimental trial, and to refrain from consuming caffeinated beverages during the 24 h before each experimental trial. In a randomized order (computer generated randomization), generated by an independent third-party researcher, participants completed two experimental trials separated with at least a 5 days washout period, to accommodate the participants’ availability. Trials for female athletes were scheduled during the follicular phase of their menstrual cycle (*n* = 5) or when taking the active medication of the oral contraceptive pill (*n* = 1). Resting estrogen levels (DKO003/RUO; DiaMetra, Italy) were measured for verification, were within normal reference range, and did not differ between trials [11.6 (6.0) pg/ml; *P* = 0.593] (Snipe and Costa, 2018b). Participants reported to the laboratory at 0800 h after consuming the standardized mixed carbohydrate breakfast [energy 2.9 (0.8) MJ, protein 28 (9) g, fat 19 (5) g, carbohydrate 99 (28) g, fiber 12 (5) g, and water 363 (264) ml] at ∼0700 h. Before commencing the exercise protocol, participants were asked to void. Pre-exercise nude BM and total body water (TBW) (Seca 515 MBCA, Seca Group, Hamburg, Germany) were recorded. Participants inserted a thermocouple 12 cm beyond the external anal sphincter to record pre-exercise rectal temperature (*T*_re_) (Precision Temperature 4600 Thermometer, Alpha Technics, CA, United States). Participants provided a breath sample into a 250 ml breath collection bag (Wagner Analysen Technik, Bremen, Germany), and completed an exercise-specific mVAS gastrointestinal symptom (GIS) assessment tool (Gaskell et al., 2019). Blood was collected by venepuncture from an antecubital vein into three separate vacutainers (6 ml 1.5 IU/ml lithium heparin, 4 ml 1.6 mg/ml K_3_EDTA, and 5 ml SST; BD, Oxford, United Kingdom). The exercise protocol consisted of a 2 h (initiated at 0900 h) high intensity interval running exercise (HIIT) session in *T*_amb_ 23.4 (1.1)°C and 44 (6)% RH, as described in Figure 1. The protocol was designed to provide sufficient exercise stress to perturb key markers of physiological and metabolic homeostasis (e.g., muscle glycogen, muscle protein, and hydration), including immune and gastrointestinal status, as previously reported (Snipe et al., 2018a; Costa et al., 2019, 2020b; Russo et al., 2019), and to mimic exertional strain incurred by continuous and intermittent endurance activities (e.g., recreational or competitive). During exercise, participants were provided with water equivalent to 3 ml/kg BM/h (Costa et al., 2009, 2011). HR (Polar Electro, Kempele, Finland), RPE, and thermal comfort rating (TCR) were measured at the 15 min mark of each 20 min cycle (Costa et al., 2014). Recovered HR and GIS were measured during the final 30 s of the 20 min cycle. Immediately post-exercise, nude BM and *T*_re_ were recorded. The recovery period commenced 30 min after the end of the exercise protocol to prepare for muscle biopsy sampling. Participants rested in a supine position in a sterile phlebotomy room for venous blood sampling followed by the first muscle biopsy thereafter. Muscle biopsy samples were taken 0 and 2 h into the recovery period. TBW was measured immediately after muscle biopsy sampling. Blood samples, nude BM and TBW were collected again at 2 and 4 h of recovery. Breath samples were collected and GIS recorded every 30 min throughout the recovery period. Total urine output was collected throughout the total recovery period. Weight of urine output was recorded at 2 and 4 h of recovery. After sampling at 2 h post-exercise, participants received a standardized recovery meal [energy 2.8 (0.7) MJ, protein 31 (8) g, fat 4 (2) g, carbohydrate 137 (32) g, fiber 9 (2) g, and water 415 (103) ml], and were instructed to consume as much as tolerable, and the total weight of the meal consumed was recorded. In addition, participants consumed a standardized evening meal after leaving the laboratory [energy 3.1 (1.4) MJ, protein 32 (14) g, fat 18 (17) g, carbohydrate 102 (50) g, fiber 19 (6) g, and water 757 (52) ml].

The following morning, participants returned to the laboratory (0800 h) to assess psychophysiological parameters and exercise performance. Due to unforeseen circumstances unrelated to the study intervention, 3 participants did not return for the second day of testing on one or both of their trials. Therefore, data for 14 participants (*n* = 9 males, *n* = 5 females) was included for analysis. A standardized mixed carbohydrate breakfast [energy 2.9 (1.0) MJ, protein 29 (12) g, fat 18 (6) g, carbohydrate 97 (37) g, fiber 11 (5) g, and water 414 (235) ml] was consumed at 0700 h. Nude body mass, TBW and GIS were recorded on arrival and again after the performance test. Before and after the performance test, participants completed measures of readiness to invest mental and physical effort, rated from 0 to 10, with higher ratings indicating greater readiness to invest effort (Duncan et al., 2012). Participants performed a 20 min running exercise bout to measure oxygen uptake and oxidation rates at four submaximal exercise intensities (50, 60, 70, and 80% *V̇*O_2__max_) for 5 min each. Thereafter, in accordance with the cohort population (recreationally trained endurance athletes and varied endurance modalities) they were asked to complete a 1 h performance test in thermoneutral conditions [*T*_amb_ 23.0 (1)°C and 46 (9)% RH]. Participants were instructed to run the maximal distance they were capable of running in 1 h, with the incline set at 1%, as previously reported (Oliver et al., 2007, 2009; Costa et al., 2017a). Total distance, HR, RPE, and water intake (provided *ad libitum*) were recorded every 10 min.

### Muscle Biopsy Procedure

Nine participants consented to providing muscle samples for both trials (*n* = 8 males, *n* = 1 female). Muscle biopsies were performed using a modified 5 mm Bergstrom biopsy needle. Samples were obtained from the vastus lateralis of the ipsilateral leg for the first trial, and contralateral leg for the second. The skin of the lateral aspect of the mid-thigh was washed well (10% Povidone- Iodine solution) then 2–3 ml of local anesthetic (lidocaine 1%) was infiltrated subcutaneously over vastus lateralis to anesthetize the skin and superficial fascia. After the anesthetic had taken effect, two 5 mm stab incisions ∼15 mm apart were made through skin and fascia, with one incision made for each muscle biopsy sample. Samples were then extracted, immediately submerged in liquid nitrogen and stored at −80°C prior to further analysis.

### Recovery Beverages

In a randomized, counterbalanced, repeated measures design, participants were provided with: (1) chocolate flavored dairy milk recovery beverage (CM) on one occasion, and a (2) orange flavored carbohydrate-electrolyte beverage (CEB) on another occasion. The beverages were prepared by a third-party researcher, and served in opaque bottles, at ∼7°C beverage temperature (Snipe and Costa, 2018a), in 3 equal boluses every 10 min, beginning 60 min after cessation of exercise. It was not possible to match the recovery beverage to taste and texture (e.g., dairy milk versus water-based solutions); however, participants were blinded to all aspects of the recovery beverage until ingestion, whereby distinct flavor and texture was apparent. The volume of the beverage was calculated to provide 1.2 g/kg BM of carbohydrate and 0.4 g/kg BM of protein on CM [energy 2449 (358) kJ, protein 28 (4) g, fat 11 (2) g, and carbohydrate 83 (12) g], and was in accordance with previous recovery research targeting muscle glycogen resynthesis, muscle protein resynthesis, and exercise-induced immunodepression prevention and (or) restoration (Russo et al., 2019). The CEB was matched for volume on the alternate trial [energy 898 (131) kJ, protein 0 (0) g, fat 0 (0) g, and carbohydrate 52 (8) g] and provided 0.76 g/kg BM of carbohydrate and 0.0 g/kg BM of protein. Additional water calculated to provide a total fluid intake of 35 ml/kg BM was provided at equal volume at hourly intervals. Participants were instructed to drink as much as tolerable. Total fluid intake was recorded hourly. The percentage of ingested fluid retained was calculated from the difference between ingested fluid and urine output, as a fraction of total fluid intake (Maughan and Leiper, 1995).

### Sample Analysis

Blood glucose concentration, hemoglobin, total and differential leukocyte counts, which included neutrophils, lymphocytes and monocytes, were determined by HemoCue system (Glucose 201+, Hb201, and WBC DIFF, HemoCue AB, Ängelholm, Sweden) in duplicate from heparin whole blood samples. Coefficient of variation (CV) for blood glucose concentration, hemoglobin, and leukocyte counts were 5.1, 1.6, and 13.6%, respectively. Hematocrit was determined by capillary method in triplicate from heparin whole blood samples and using a microhematocrit reader (CV: 0.7%) (Thermo Fisher Scientific). Hemoglobin and hematocrit values were used to estimate changes in plasma volume (P_V_) relative to baseline, and used to correct plasma variables (Dill and Costill, 1974). To determine the blood glucose response to the recovery beverage, immediately before and every 30 min thereafter for 2 h, blood glucose concentration was measured in duplicate using a handheld system from capillary blood samples (CV: 4.1%) (Accu-Chek *Proforma*, Roche Diagnostics, Indianapolis, IN, United States). To determine *in vitro* bacterially-stimulated elastase release, 1 ml of whole blood was pipetted into a microcentrifuge tube containing 50 μg of 1 mg/ml bacterial stimulant (lipopolysaccharide from *Escherichia coli*, Sigma, Poole, United Kingdom) within 5 min of collection and gently vortex-mixed. Samples were incubated in a water bath (Labline, Thermo Fisher Scientific Australia, Scoresby, VIC, Australia) at 37°C for 1 h, and further mixed by gentle inversion at 30 min. Bacterially challenged samples were then centrifuged at 4,000 rpm (1,500 *g*) for 10 min, and supernatant was aspirated into 1.5 ml micro-storage tubes and stored at −80°C for further analysis. The remaining whole blood in the heparin and K_3_EDTA vacutainers were centrifuged at 4,000 rpm (1,500 *g*) for 10 min within 15 min of sample collection. The whole blood collected in the SST serum tube was allowed to clot for 1 h in ∼4°C prior to centrifuging at 4,000 rpm (1,500 *g*) for 10 mins. 2 × 50 μl of heparin plasma was used to determine plasma osmolality (*P*_Osmol_), in duplicate (CV: 0.7%), by freeze point osmometry (Osmomat 030, Gonotec, Berlin, Germany). The remaining heparin and K_3_EDTA plasma, and SST serum was aspirated into the appropriate 1.5 ml micro-storage tubes and frozen at −80°C until analysis. Circulating concentrations of insulin (DKO076; DiaMetra, Italy), cortisol (DKO001; DiaMetra, Italy), aldosterone (Demeditec Diagnostics GmbH, Kiel, Germany), PMN elastase (BMS269; Affymetrix EBioscience, Vienna, Austria), intestinal fatty acid-binding protein (I-FABP) (HK406; Hycult Biotech, Uden, Netherlands), sCD14 (HK320; Hycult Biotech), and lipopolysaccharide binding protein (LBP) (HK315; Hycult Biotech) were determined by ELISA. Additionally, systemic cytokine profile (including plasma interleukin (IL)-1β, tumor necrosis factor (TNF)-α, IL-6, IL-8, IL-10, and IL-1 receptor antagonist (ra) concentrations) (HCYTMAG-60K, EMD Millipore, Darmstadt, Germany) were determined by multiplex system. All variables were analyzed as per manufacturer’s instructions on the same day, with standards and controls on each plate, and each participant assayed on the same plate. The CVs for ELISAs were ≤6.1% and for cytokine profile multiplex was 16.0%. Breath samples (20 ml) were analyzed in duplicate (CV: 2.1%) for hydrogen (H_2_) content using a gas-sensitive analyzer (Breathtracker Digital Microlyzer, Quintron, Milwaukee, WI, United States). Plasma sodium, potassium and calcium concentrations were determined using ion selective electrodes (Cobas c analyzer, Roche Diagnostics, Risch-Rotkreuz, Switzerland) and analyzed by local pathology services (Cabrini Pathology, Malvern, VIC, Australia).

### Western Blot Analysis

Approximately 30 mg of skeletal muscle was solubilized in radioimmunoprecipitation (RIPA) buffer (Millipore, Bayswater, VIC, Australia) with 1 μl/ml protease inhibitor cocktail (Sigma-Aldrich, Castle Hill, NSW, Australia) and 10 μl/ml Halt Phosphatase Inhibitor Single-Use Cocktail (Thermo Scientific, Australia, North Ryde, NSW, Australia). The concentration of protein per sample was determined by the bicinchoninic acid assay (BCA Protein Assay Kit#23225, Thermo Scientific). 20 μg of skeletal muscle protein lysate was loaded onto into either Bio-Rad precast Criterion TGX Stain-Free 4–12% gels (Bio-Rad, Gladesville, NSW, Australia). SDS-PAGE was conducted following manufacturer’s instructions. Protein was then transferred to PVDF membranes and blocked for 1 h in 5% bovine serum albumin (BSA) solution in Tris-buffered saline-Tween, (pH 7.6, 20 mmol/L Tris and 150 mmol/L NaCl, 0.1% Tween) (TBST) at room temperature. Membranes were then incubated in primary antibodies diluted in 5% BSA/TBST overnight at 4°C. Following washing in TBST, membranes were incubated for 1 h with fluorescent secondary antibodies [phospho-mammalian target of rapamycin (mTOR)^Ser2448^, phospho-protein kinase B (Akt)^Ser473^, phospho-ribosomal protein S6 (rpS6)^Ser235/236^, and phosphor-glycogen synthase kinase 3β (GSK-3β)^Ser9^] [anti-Rabbit IgG (H + L) Dylight^TM^ 800 Conjugate; Anti-mouse IgG (H + L) Dylight^TM^ 680 Conjugate] (Cell Signalling Technologies^®^, Danvers, MA, United States) diluted 1:10,000 in TBST. Following 2 further washes in TBST and 1 wash in phosphate buffered saline (PBS) membranes were scanned using the LiCOR^®^ Odyssey CLx^®^ Imaging System (Millennium Science, Mulgrave, VIC, Australia). All targets were normalized to total protein using either the Bio-Rad stain-free system.

### Muscle Glycogen Analysis

One fraction of muscle sample [approx. 20–25 mg (ww)] was freeze-dried, after which collagen, blood and other non-muscle material were removed from the muscle fibers. Samples were then pulverized and powdered. Samples were extracted with 0.5 M perchloric acid (HClO_4_) containing 1 nmol EDTA and neutralized using 2.2 M KHCO_3_. Adenosine triphosphate, phosphocreatine, and creatine was determined from the supernatant by enzymatic spectrophotometric assays (Bergmeyer, 1974; Harris et al., 1974). Muscle glycogen content was determined from 2 aliquots of freeze-dried muscle (2–3 mg), as previously reported (Bergmeyer, 1974).

### Statistical Analysis

Confirmation of adequate statistical power was determined *a priori* from the applied statistical test, mean, standard deviation, and effect size on markers of (1) gastrointestinal integrity (i.e., plasma I-FABP), function (i.e., breath hydrogen), and GIS (Costa et al., 2017a, 2019; Snipe et al., 2018a, b); (2) circulating leukocyte, endotoxin and cytokine profiles (Costa et al., 2009, 2011, 2019, 2020a); (3) total body water, plasma osmolality, plasma volume change (Costa et al., 2019, 2020a); (4) rate of skeletal muscle glycogen resynthesis (Betts et al., 2007; Alghannam et al., 2016); (5) phosphorylation of intramuscular signaling proteins (Camera et al., 2015, 2016); and (6) performance (Oliver et al., 2007, 2009). Using a standard alpha (0.05) and beta value (0.80), the current participant sample size, within a repeated measures cross-over design, is estimated to provide adequate statistical power (power^∗^ 0.80–0.99) for detecting significant between- (trial) and within- (time) group differences (G^∗^Power 3.1, Kiel, Germany). Data in the text and tables are presented as mean (SD) for descriptive method, and mean and 95% confidence interval (CI) for primary variable, as indicated. For clarity, data in figures are presented as mean and standard error of the mean (SEM), and (or) mean and individual responses, as indicated. Systemic inflammatory cytokine responses are presented as raw values and systemic inflammatory response profile (SIR-profile), as previously reported (Bennett et al., 2020). Only participants with full data sets within each specific variable were used in the data analysis. There were no outliers for female participant data points for any of the primary and secondary outcome measures. All data were checked for normal distribution (i.e., Shapiro–Wilks test of normality) prior to main within- and between-group comparative analysis. Variables with singular data points were examined using paired sample *t*-tests, or non-parametric Wilcoxon signed-rank test, when appropriate. Variables with multiple data points were examined using a two-way repeated-measures ANOVA. Assumptions of homogeneity and sphericity were checked, and when appropriate adjustments to the degrees of freedom were made using the Greenhouse–Geisser correction method. Main effects were analyzed by Tukey’s *post hoc* HSD. Statistics were analyzed using SPSS statistical software (V.26.0, IBM SPSS Statistics, IBM Corp., Armonk, NY, United States) with significance accepted at *P* ≤ 0.05. Additionally, Hedge’s g measurement of effect size for GIS and feeding tolerance severity between CM and CEB was determined as >0.50 and >0.80 for medium and large effects, respectively.

## Results

### Exertional Strain

During exercise, a main effect of time (MEOTime) was observed for peak [overall mean and 95% CI: 157 (155–159) bpm; *P* < 0.001] and recovered HR [119 (117–121) bpm; *P* < 0.001], RPE [13 (13–13); *P* < 0.001], and TCR [9 (8–9); *P* = 0.005]; whereby HR, RPE, and TCR increased as exercise progressed on CM and CEB, with no differences between trials observed, *T*_re_ increased pre- [36.8 (36.6–37.0)°C] to post-exercise [37.9 (37.7–38.1)°C] on CM and CEB (*P* < 0.001), with no difference between trials observed. A trial^∗^time interaction was observed for plasma cortisol concentration (*P* = 0.012) (Table 1), whereby Δ pre- to post-exercise plasma cortisol concentrations was greater on CEB [274 (109–439) nmol/L] compared with CM [53 (−92–197) nmol/L]. However, no trial differences were observed during the recovery period. Exercise-induced BM loss [1.9 (1.7–2.2)%], Δ*P*_V_ [−359 3.1 (−5.0 to −1.2)%], pre- and post-exercise *P*_Osmol_ and TBW (including extracellular and intracellular water) did not differ between CM and CEB (Table 1).

**TABLE 1:** Change in hydration and recovery optimization biomarkers in response to 2 h HIIT exercise (between 60 and 80% *V̇*O_2__max_) in temperate ambient conditions and after consumption of dairy milk (CM) and carbohydrate-electrolyte (CEB) recovery beverages.

	CM				CEB			
	Pre-exercise	Post-exercise	2 h post-exercise	4 h post-exercise	Pre-exercise	Post-exercise	2 h post-exercise	4 h post-exercise
Total body water (%)	59.8 (57.6–62.0)	60.2 (58.0–62.3)	59.3^††^ (57.2–61.4)	59.2^††^ (57.1–61.3)	59.5 (57.5–61.6)	60.4 (58.1–62.7)	58.9^††^ (56.8–61.1)	59.2^††^ (57.1–61.2)
(L)	40.6 (37.8–43.4)	40.2 (37.3–43.2)	40.2^§§^ (37.4–43.1)	40.6 (37.8–43.4)	40.4 (37.7–43.1)	40.2 (37.5–43.0)	39.8^§§^ (37.0–42.6)	40.3 (37.5–43.0)
Extracellular water (%)	24.5 (23.8–25.2)	24.4 (23.7–25.1)	24.1^§§††^ (23.4–24.8)	24.1^§§††^ (23.4–24.8)	24.4 (23.7–25.1)	24.3 (23.6–25.0)	23.9^§§††^ (23.3–24.6)	24.0^§§††^ (23.4–24.7)
(L)	16.8 (15.8–17.9)	16.4^§§^ (15.4–17.5	16.4^§§^ (15.4–17.5)	16.6^§†^ (15.6–17.7)	16.6 (15.6–17.6)	16.2^§§^ (15.3–17.2)	16.2^§§^ (15.3–17.2)	16.4^§†^ (15.4–17.4)
*P*_Osmol_ (mOsmol/kg)	292 (288–296)	296 (291–301)	293 (290–296)	292^†^ (289–296)	290 (287–293)	293 (289–296)	289 (285–292)	288^†^ (284–291)
Δ *P*_V_ (%)	–	−4.8^§§^ (−7.6–−1.9)	−1.1^††^ (−4.0–1.8)	−0.1^††^ (−2.3–2.2)	–	−2.3^§§^ (−3.7–0.9)	+3.7^††^ (1.5–6.0)	+3.6^††^ (0.2–7.0)
Cortisol (nmol/L)	349 (305–594)	461 (348–575)	296*^##^ (216–377)	220**^##^ (133–307)	449 (304–593)	716**^aa^ (544–888)	340^##^ (235–446)	236**^##^ (148–325)
I−FABP^‡^ (pg/ml)	616 (435–796)	1244^§§^ (862–1626)	–	–	494 (363–625)	848^§§^ (647–1049)	–	–
sCD14 (μg/ml)	2.21 (1.9–2.52)	2.05 (1.72–2.39)	–	–	2.42 (2.28–2.56)	2.53 (2.38–2.67)	–	–
LBP (μg/ml)	11.5 (7.9–15.2)	11.6 (8.5–14.8)	–	–	12.0 (0.7–15.3)	13.2 (9.1–17.3)	–	–
IL-1β (pg/ml)	3.8 (1.3–6.3)	4.0 (1.7–6.3)	4.0 (1.7–6.4)	4.7 (2.0–7.5)	2.8 (1.2–4.4)	2.8 (1.7–4.0)	2.6 (1.1–4.1)	3.1 (1.2–4.9)
TNF-α (pg/ml)	14.9 (10.5–19.3)	14.4 (10.3–18.5)	14.3 (9.8–18.9)	17.3 (10.5–24.0)	11.4 (8.8–14.1)	13.9 (10.2–17.6)	12.9 (9.7–16.1)	14.0 (8.5–19.5)
IL-6 (pg/ml)	37.3 (<1.0^c^–75.7)	40.1 (<1.0^c^–79.7)	41.3 (<1.0^c^–83.0)	34.8 (<1.0^c^–69.1)	35.7 (<1.0^c^–72.5)	33.9 (<1.0^c^–69.1)	34.8 (<1.0^c^–70.9)	33.4 (<1.0^c^–68.4)
IL-8 (pg/ml)	19.1 (3.4–34.8)	20.1 (4.2–35.9)	19.4 (3.2–35.7)	18.2 (4.5–31.9)	16.2 (1.5–30.9)	17.7 (4.2–31.2)	16.9 (2.2–31.6)	16.5 (2.0–31.0)
IL-10 (pg/ml)	25.5 (13.2–37.7)	34.2^§^ (20.8–47.6)	23.0^†^ (13.1–32.8)	24.1^†^ (12.7–35.4)	18.2 (10.8–25.7)	34.7^§^ (22.1–47.2)	17.3^†^ (11.5–23.1)	14.8^†^ (9.2–20.4)
IL-1rα (pg/ml)	37.0 (21.2–52.8)	45.7 (27.6–63.7)	52.9^§^ (33.6–72.2)	44.2 (30.9–57.5)	37.7 (20.6–54.9)	39.8 (26.7–52.9)	44.4^§^ (29.0–59.8)	44.0 (29.6–58.4)
Aldosterone (nmol/L)	96 (67–125)	292^§§^ (213–371)	100^††^ (79–121)	85^††^ (61–110)	130 (92–168)	462^§§^ (292–633)	132^††^ (84–180)	111^††^ (63–158)
Serum sodium (mmol/L)	140 (139–141)	134 (130–138)	138^†^ (134–142)	139^††^ (136–143)	139 (137–141)	135 (133–138)	145^†^ (141–148)	143^††^ (139–148)
Serum potassium (mmol/L)	4.2 (3.6–4.8)	4.2 (3.7–4.8)	4.1 (3.5–4.7)	4.3 (3.7–4.9)	4.6 (4.4–4.9)	4.3 (4.1–4.6)	4.6 (4.3–5.0)	4.4 (4.1–4.7)
Serum calcium (mmol/L)	2.34 (2.31–2.37)	2.17^§§^ (2.10–2.23)	2.31^††^ (2.24–2.39)	2.32^††^ (2.26–2.37)	2.32 (2.29–2.35)	2.21^§§^ (2.17–2.25)	2.38^††^ (2.32–2.43)	2.38^††^ (2.29–2.46)

Mean (95% CI) (n = 17): MEOTrial ^‡^P < 0.05, MEOTime ^§§^P < 0.01 and ^§^P < 0.05 vs. pre-exercise, MEOTime ^††^P < 0.01 and ^†^P < 0.05 vs. post-exercise, trial*time interaction **P < 0.01 and *P < 0.05 vs. pre-exercise, trial*time interaction ^##^P < 0.01 and ^#^P < 0.05 vs. post-exercise, ^aa^P < 0.01 and ^a^P < 0.05 vs. CM, ^c^under detectable lowest standard.

### Gastrointestinal Integrity, Function and Symptoms

**FIGURE 2:**
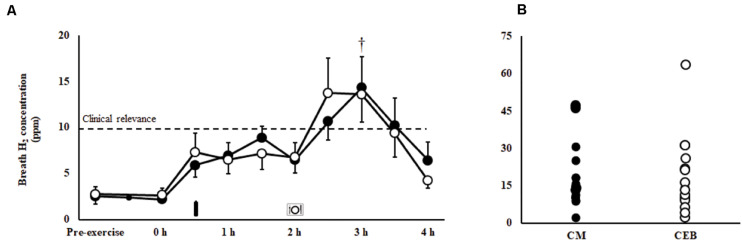
Breath hydrogen response **(A)** and individual peak breath hydrogen **(B)** after 2 h HIIT exercise in temperate ambient conditions and consumption of a chocolate dairy milk (CM: ■) or the carbohydrate-electrolyte (CEB: ∘) recovery beverage. Mean ± SEM (*n* = 17): MEOTime ^†^*P* < 0.05 vs. post-exercise.

A MEOTrial (*P* = 0.020) and MEOTime (*P* = 0.003) were observed for plasma I-FABP concentration, whereby levels were generally higher pre- and post-exercise on CM, and increased in response to the exercise stress on both trials; however, magnitude of response was not significantly different between trial (Table 1). No significant main effects or interactions were observed for plasma sCD14 or LBP concentrations (Table 1). There were no significant differences in the incidence or severity of GIS during exercise and recovery period between (Table 2). There was a MEOTime for breath H_2_ (*P* < 0.001), a peak of clinical significance [CM: 17 (11–24) ppm vs. CEB: 18 (11–25) ppm] occurring following consumption of both recovery beverage (Figure 2).

**TABLE 2:** Incidence of gastrointestinal symptoms and severity of gut discomfort, total, upper-, and lower-gastrointestinal symptoms in response to 2 h HIIT exercise (between 60 and 80% *V̇*O_2__max_) in temperate ambient conditions and after consumption of dairy milk (CM) and the carbohydrate-electrolyte (CEB) recovery beverages.

	CM					CEB				
	Exercise		Recovery			Exercise		Recovery		
	Incidence	Severity	Incidence	Severity		Incidence	Severity	Incidence	Severity	
	% (severe)		% (severe)	Acute (1–2 h)	Total (1–4 h)	% (severe)		% (severe)	Acute (1–2 h)	Total (1–4 h)
**Gut discomfort**	NA	5 (0–13)	NA	5 (0–24)	14 (0–51)	NA	5 (0–18)	NA	4 (0–22)	11 (1–50)
**Total GIS^1^**	59 (0)	6 (4–18)	76 (47)	6 (3–24)	15 (3–51)	71 (12)	7 (2–31)	64 (35)	4 (1–22)	12 (1–50)
**Upper GIS^2^**	47 (0)	3 (3–10)	41 (29)	4 (10–24)	11 (2–51)	35 (0)	2 (1–11)	52 (24)	3 (1–22)	8 (2–50)
Belching	35 (0)	3 (3–10)	18 (0)	0 (1–1)	0 (1–2)	29 (0)	2 (1–11)	18 (0)	0 –	1 (2–4)
Heartburn	6 (0)	0 (3–3)	6 (0)	0 –	0 (4–4)	0 (0)	0 –	0 (0)	0 –	0 –
Bloating	12 (0)	0 (2–4)	35 (29)	4 (10–24)	10 (2–51)	12 (0)	0 (1–3)	41 (24)	3 (1–22)	7 (3–50)
Stomach pain	0 (0)	0 –	0 (0)	0 –	0 –	0 (0)	0 –	0 (0)	0 –	0 –
Urge to regurgitate	6 (0)	0 (1–1)	0 (0)	0 –	0 –	0 (0)	0 –	0 (0)	0 –	0 –
Regurgitation	0 (0)	0 –	0 (0)	0 –	0 –	0 (0)	0 –	0 (0)	0 –	0 –
**Lower GIS^2^**	24 (0)	1 (1–6)	42 (18)	1 (3–8)	3 (3–20)	18 (0)	1 (7–13)	35 (12)	1 (5–10)	3 (1–25)
Flatulence	12 (0)	0 (1–3)	12 (0)	0 –	0 (3–5)	18 (0)	1 (7–13)	24 (0)	0 –	1 (1–7)
Lower bloating	6 (0)	0 (2–2)	18 (0)	0 –	1 (2–11)	6 (0)	0 –	6 (6)	0 –	0 (8–8)
Urge to defecate	12 (0)	0 (1–3)	24 (0)	0 –	2 (2–12)	0 (0)	0 –	12 (6)	1 (5–10)	1 (5–10)
Intestinal pain	0 (0)	0 –	0 (0)	0 –	0 –	0 (0)	0 –	0 (0)	0 –	0 –
Abnormal defecation^3^	0 (0)	0 –	6 (6)	0 (6–6)	0 –	0 (0)	0 –	6 (6)	0 –	0 (10–10)
**Others**										
Nausea	12 (0)	1 (1–11)	6 (0)	0 (5–5)	0 (5–5)	24 (6)	1 (1–12)	12 (0)	1 (4–7)	1 (4–7)
Dizziness	29 (0)	2 (2–12)	24 (0)	1 (1–7)	1 (1–7)	29 (6)	1 (1–11)	18 (0)	0 (1–3)	0 (1–3)
Stitch^4^	6 (0)	0 (1–1)	0 (0)	0 –	0 –	6 (0)	0 (4–4)	0 (0)	0 –	0 –
**Feeding tolerance**										
Appetite	NA	13 (0–39)	NA	21 (3–35)	26 (5–43)	NA	14 (0–38)	NA	25 (8–43)	32^a^ (9–58)
Thirst	NA	23 (0–44)	NA	16 (4–26)	21 (6–37)	NA	23 (0–41)	NA	19 (8–37)	26 (14–51)

*Values are presented as means and range of participant reporting GIS incidence (n = 17). GIS incidence during exercise and recovery are presented as percentage of total participants reporting GIS ≥ 1 on the mVAS. GIS severity during exercise and recovery are presented as mean summative accumulation of mVAS rating scale of measured time periods and individual range of participant reporting GIS incidence (Gaskell et al., 2019, 2020b). ^1^Summative accumulation of upper-, lower-, and other gastrointestinal symptoms, ^2^summative accumulation of upper- or lower- gastrointestinal symptoms, ^3^abnormal defecation including loose watery stools, diarrhea and blood in stools, and ^4^acute transient abdominal pain. NA, not applicable. Wilcoxon signed-rank tests showed no differences between CM and CEB for GIS; however ^a^ p < 0.05 vs. CM for appetite. Hedge’s g measurement of effect size for GIS and feeding tolerance severity between CM and CEB was determined as >0.50 and >0.80 for medium and large effects, respectively; however, no medium or large effects size value were detected between CM and CEB.*

### Glucose Availability and Insulin Response

**FIGURE 3:**
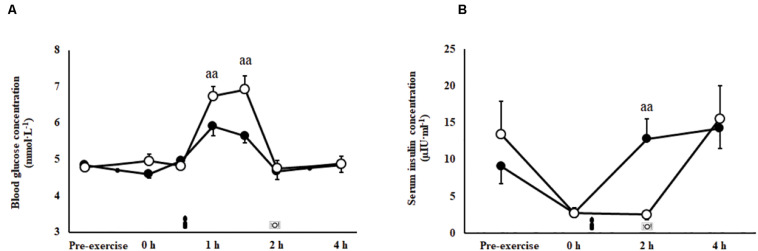
Blood glucose **(A)** and serum insulin **(B)** concentrations after 2 h HIIT exercise protocol in temperate ambient conditions and consumption of a chocolate dairy milk (CM: ■) or the carbohydrate-electrolyte (CEB: ∘) recovery beverage. Mean ± SEM (*n* = 17): ^aa^*P* < 0.01 vs. CM.

A trial^∗^time interaction was observed for blood glucose (*P* = 0.001; Figure 3A) and serum insulin responses (*P* = 0.007; Figure 3B) imposed by feeding in the recovery period. Peak blood glucose concentration (*P* = 0.001) and area under the curve (*P* = 0.001) were greater during the 2 h after consumption of CEB [7.5 (6.9–8.0) mmol/L and 700 (665–735) mmol/L/2 h, respectively] compared with CM [6.3 (5.8–6.7) mmol/L and 634 (601–667) mmol/L/2 h, respectively]. Insulin levels were significantly greater at 2 h recovery following consumption of CM compared with CEB (*P* < 0.01).

### Immune Responses

**FIGURE 4:**
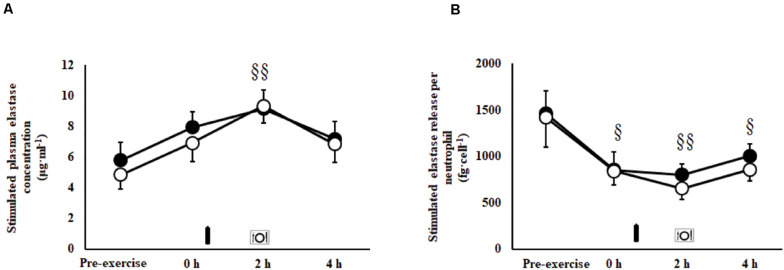
Total **(A)** and per cell **(B)** bacterially-stimulated neutrophil elastase release after 2 h HIIT exercise in temperate ambient conditions and consumption of a chocolate dairy milk (CM: ■) or the carbohydrate-electrolyte (CEB: ∘) recovery beverage. Mean ± SEM (*n* = 14): MEOTime ^§§^*P* < 0.01 and ^§^*P* < 0.05 vs. pre-exercise.

An exercise-induced leukocytosis [10.5 (9.6–11.4) × 10^9^/L; *P* < 0.001], neutrophilia [7.4 (6.5–8.2) × 10^9^/L; *P* < 0.001], lymphocytosis [2.9 (2.5–3.4) × 10^9^/L; *P* < 0.001], monocytosis [0.6 (0.6–0.7) × 10^9^/L; *P* < 0.001], and increased neutrophil:lymphocyte ratio [3.3 (2.9–3.8); *P* < 0.001] were observed in the recovery period on both trials. No main effects or interaction were observed for unstimulated plasma elastase concentration [146 (124–170) ng/ml]. A MEOTime was observed for total bacterially-stimulated plasma elastase concentration (*P* = 0.003; Figure 4A), and bacterially-stimulated elastase release per neutrophil (*P* = 0.015; Figure 4B). Whereby, bacterially-stimulated total elastase release increased (71%) during recovery, peaking at 2 h post-exercise in both trials; while relative per neutrophil elastase release values decreased (49%), toughing at 2 h post-exercise in both trials. A MEOTime was observed for plasma IL-10 (*P* = 0.006) and IL-1ra (*P* = 0.016) concentrations; whereby the exercise protocol induced compensatory anti-inflammatory responses in recovery. No main effects or interaction were observed for plasma IL-1β, TNF-α, IL-6, and IL-8 concentrations. No difference in exercise-induced SIR-profile [CM: 45 (26–64) arb.unit and CEB: 40 (22 to 58) arb.unit] and recovery beverage post-prandial SIR-profile [CM: 11 (−9 to 30) arb.unit and CEB: −1 (−21 to 18) arb.unit] were observed.

### Muscle Glycogen

**FIGURE 5:**
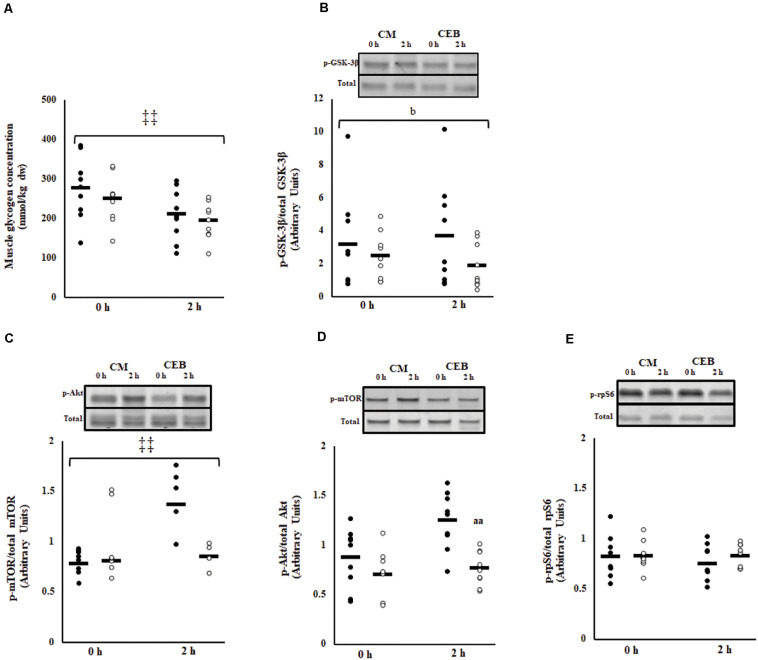
Muscle glycogen content **(A)**, phosphorylated GSK3-β to total GSK3-β **(B)**, ratio of phosphorylated mTOR to total mTOR **(C)**, phosphorylated Akt to total Akt **(D)**, and phosphorylated rpS6 to total rpS6 **(E)** after 2 h HIIT exercise in temperate ambient conditions and consumption of a chocolate dairy milk (CM: ■) or the carbohydrate-electrolyte (CEB: □) recovery beverage. Mean and individual responses (*n* = 9): MEOTrial ^‡‡^ *P* < 0.01, ^b^*P* < 0.05 vs. CM in magnitude, ^aa^*P* < 0.01 vs. CM.

Post-exercise muscle glycogen content was 262 (230–294) mmol/kg dw (Figure 5A). The early rate of muscle glycogen formation did not differ between trials [−30.3 (−39.4 to −21.2) mmol/kg dw/h]; however, muscle glycogen concentrations were higher on CM (*P* < 0.001). No main effects or interaction were observed for the ratio of phosphorylated GSK-3β to total GSK-3β; however, the fold change was significantly greater on CM [1.3 (0.9–1.6)] compared with CEB [0.8 (0.6–0.9] (*P* = 0.037) (Figure 5B).

### Phosphorylation of Muscle Signaling Proteins

The ratio of phosphorylated mTOR to total mTOR (p-mTOR/TOTAL mTOR) increased after consumption of CM, but not after consumption of CEB (time^∗^trial, *P* < 0.001) (Figure 5C). The overall ratio of phosphorylated Akt to total Akt (p-Akt/Akt) was higher on CM (*P* < 0.001); however, there was no MEOTime and the magnitude of change from 0 to 2 h recovery did not differ between recovery beverages (Figure 5D). No main effects or interaction were observed for phosphorylation of rpS6 (Figure 5E).

### Hydration and Plasma Electrolyte Status

Total fluid intake during the recovery period [CM: 22.9 (20.7–25.1) ml/kg BM and CEB: 22.1 (19.6–24.6) ml/kg BM] did not differ between trials. There was a MEOTime imposed by the rehydration intervention for *P*_Osmol_ (*P* = 0.004), P_V_ (*P* < 0.001), rating of thirst (*P* < 0.001), and TBW (*P* < 0.001) including extracellular (*P* < 0.001) and intracellular (*P* < 0.001) water, with values returning to near baseline values 2 h into the recovery period (Table 1). *P*_V_ was higher in the recovery period on CEB compared with CM (*P* = 0.015; Table 1). This was in adjunct with total urine output being higher (*P* = 0.028) and total fluid retention being lower (*P* = 0.040) on CEB [393 (286–500) ml and 80.2 (75.9–84.5)%, respectively] compared with CM [279 (217–341) ml and 85.8 (82.5–89.0)%, respectively]. A MEOTime (*P* < 0.001) was observed for plasma aldosterone concentration; whereby levels increase in response to exercise and reduced back to baseline during recovery, with no differences associated with consumption of the different recovery beverages (Table 1). A MEOTime was observed for plasma sodium (*P* = 0.001) and calcium (*P* = 0.001) concentrations (Table 1). Plasma calcium concentration decreased in response to exercise (*P* < 0.01). Both plasma calcium and sodium increased from post-exercise to 2 and 4 h recovery, with no differences associated with consumption of the different recovery beverages. No main effects or interaction were observed for plasma potassium concentration (Table 1).

### Psychophysiological Parameters and Performance Outcomes

Participants reported greater readiness of invest mental (*P* = 0.006) and physical effort (*P* = 0.001) to perform following consumption of CM [6 (5–6) and 5 (4–6), respectively] compared to CEB [5 (4–6) and 4 (3–5), respectively]. A greater decrease in physical readiness to perform was observed on CEB pre- to post-performance testing (*P* = 0.014). There were no differences between treatments in pre-exercise nude BM [69.3 (65.8–72.8) kg] or TBW [60 (58–61)%] the morning after the exercise trial. There were no trial differences in carbohydrate and fat oxidation rates or physiological parameters during the incremental test (Table 3). Mean HR [165 (163–168) bpm], RPE [15 (15–16)] and water intake [430 (328–532) ml] did not differ across the distance test between trials. Total distance run over 1 h did not differ between CM [12 (11.2–12.9) km] and CEB [11.9 (10.9–12.9) km].

**TABLE 3:** Physiological and performance outcomes of graded exercise intensity using breath-by-breath testing between 50–80% *V̇*O_2__max_ and 1 h self-paced distance performance test, following consumption of dairy milk (CM) and carbohydrate-electrolyte (CEB) recovery beverages the previous day.

		CM			CEB			
	50% *V̇*O_2max_	60% *V̇*O_2max_	70% *V̇*O_2max_	80% *V̇*O_2max_	50% *V̇*O_2max_	60% *V̇*O_2max_	70% *V̇*O_2max_	80% *V̇*O_2max_
RER	0.88 (0.86–0.90)	0.91 (0.89–0.93)	0.93 (0.91–0.94)	0.97 (0.96–0.99)	0.88 (0.85–0.90)	0.91 (0.88–0.94)	0.93 (0.90–0.96)	0.97 (0.95–0.99)
*V̇*O_2_ (ml/kgBM/min)	28.3 (25.6–30.9)	33.7 (30.5–36.9)	41.2 (38.3–44.1)	47.5 (44.2–50.8)	27.1 (25.0–29.3)	32.8 (30.2–35.5)	39.9 (37.3–42.6)	46.4 (43.5–49.4)
Carbohydrate oxidation (g/min)	1.62 (1.31–1.93)	2.22 (1.91–2.53)	2.93 (2.57–3.29)	4.09 (3.67–4.52)	1.54 (1.22–1.85)	2.18 (1.77–2.59)	2.94 (2.45–3.43)	3.99 (3.43–4.54)
Fat oxidation (g/min)	0.38 (0.31–0.45)	0.34 (0.26–0.41)	0.33 (0.27–0.40)	0.14 (0.08–0.20)	0.38 (0.29–0.46)	0.33 (0.22–0.45)	0.31 (0.20–0.43)	0.15 (0.08–0.22)
HR	116 (110–122)	133 (124–141)	150 142–158)	165 (158–172)	117 (112–123)	133 (124–141)	150 (144–157)	164 (158–171)
RPE	8 (8–9)	10 (9–11)	12 (12–13)	15 (14–16)	8 (8–9)	10 (9–11)	12 (12–13)	15 (14–15)

*Mean (95% CI) (n = 14). BM, body mass; HR, heart rate; RER, respiratory exchange ratio; RPE, rating of perceived exertion.*

## Discussion

The current study aimed to investigate the impact of a carbohydrate- and protein-containing flavored dairy beverage and a non-nitrogenous carbohydrate electrolyte beverage on overall and integrative markers of acute recovery (i.e., EIGS, immune function, muscle glycogen resynthesis, protein synthesis, rehydration, and subsequent performance outcomes the following day), after an exercise stress model known to perturb aspects of physiological and metabolic homeostasis. Carbohydrate malabsorption and GIS was evident during the recovery period on both CM and CEB. A more rapid rate of glucose availability and corresponding acute insulin response were observed on CEB, compared with CM. Post-exercise leukocyte trafficking, depressed neutrophil function in response to bacterial challenge, and modest systemic inflammatory responses were not affected by the different recovery beverages. The greater carbohydrate content of CM did not result in greater muscle glycogen resynthesis in the acute timeframe, but did result in a greater increase in phosphorylation of GSK-3β compared with CEB. Greater phosphorylation of mTOR and absolute Akt protein signaling were observed on CM compared with CEB; but no differences in rpS6 signaling protein was observed between the recovery beverages. Despite lower sodium content, greater fluid retention was observed following consumption of the carbohydrate-protein CM beverage compared to the carbohydrate-only CEB beverage. Although subjects reported an enhanced readiness to invest in physical and mental effort on the following day with CM compared to CEB, this did not translate into differences in performance outcomes on a distance test. This was presumably due to the resumption of normal dietary habits after 2 h recovery and no overall differences in 24 h nutritional intake. These findings build on our current understanding of recovery nutrition to consider acute functionality and patency of the gastrointestinal tract, and restoration of immunocompetency. As such, it is recommended that athletes consume small, frequent boluses of a protein-carbohydrate beverage immediately following cessation of exercise, in order to minimize gastrointestinal burden, support clearance and turnover of damaged epithelial and skeletal muscle tissue, and aid fluid retention.

### Gastrointestinal Response to Recovery Nutrition

It has recently been acknowledged that the epithelial injury, sympathetic drive, and associated GIS, as a result of EIGS, may compromise nutrient ingestion, digestion and (or) absorption during the exercise recovery period (Lang et al., 2006; van Wijck et al., 2013; Costa et al., 2016, 2017a, 2019, 2020a). A substantial increase in post-exercise breath H_2_, indicative of carbohydrate malabsorption of clinical significance (Bate et al., 2010), occurred on both CM and CEB, despite the vastly different nutritional profiles (e.g., carbohydrate content and types) of the recovery beverages, and evidence of heterogeneous exercise-associated intestinal epithelial injury [overall mean (95% CI): Δ I-FABP 491 (256–727) pg/ml] and stress hormone response [Δ cortisol 163 (39–288) nmol/L]. Previous literature has observed similar peak breath H_2_ values (i.e., 10–20 ppm) amongst healthy men and women following consumption of sucrose (<100 g) and lactose (50 g) at rest (Rumessen and Gudmand-Høyer, 1986; Barrett et al., 2009), suggesting a timing and bolus volume induced response. A subset of participants in the current study experienced peak breath H_2_ values >20 ppm following consumption of one or both beverages. These data suggests a wide individual variability in gastrointestinal absorptive capacity of carbohydrate, possibly linked with: (a) intestinal enterocyte carbohydrate transporter saturation capacity; and (or) (b) exercise- associated impairment of carbohydrate transporter translocation and activity effectiveness at the enterocyte brush border, secondary to increased enterocyte damage (i.e., circulatory-gastrointestinal pathway of EIGS) and (or) sympathetic activation (i.e., neuroendocrine-gastrointestinal pathway of EIGS) (Costa et al., 2017a, b). In regards to assessing protein malabsorption, previous research has suggested protein and amino acid provision immediately after exercise is not malabsorbed to any great extent, even with a wide range of protein provisions (e.g., 0.2–0.6 g/kgBM) (Mazzulla et al., 2017; Churchward-Venne et al., 2020). It was not possible within the current protocol to assess protein malabsorption using previously reported methodologies (e.g., L-[1-13C] phenylalanine labelled protein ingestion with continuous intravenous L-[ring-2H5] phenylalanine infusion) (van Wijck et al., 2013), as these would interfere with the accuracy and reliability of the primary variables of the current study. Therefore, considering the previous research, reporting (van Wijck et al., 2013) and not reporting (Mazzulla et al., 2017; Churchward-Venne et al., 2020) impairment in protein absorption of recovery nutrition provisions, and did not use an exercise stress model (e.g., resistance exercise, 90 min cycling at 60% *W*_max_, and 60 min running at 70% *V̇*O_2__peak_, all in temperate ambient conditions) sufficient to induce EIGS (Costa et al., 2017b, 2020a); it is still unknown the full impact of prolonged strenuous exercise on recovery beverage protein malabsorption, and whether any reduced protein bioavailability has any influence on cellular recovery responses.

Similar incidences and types of GIS were observed in recovery on CM (76%) and CEB (64%), with overall mild severity reaching a total varying range of 3–51 and 1–46, respectively. In addition, CM resulted in lower appetite only 4 h into recovery, likely attribute to the overall nutrient load (i.e., recovery beverage, plus recovery meal at 2 h post-exercise), inclusive of the protein dose. Previous studies have demonstrated transiently reduced appetite and feeding tolerance, and (or) increased fullness, with no greater gastrointestinal discomfort, after consumption of carbohydrate-protein versus carbohydrate and (or) non-nutritive beverages (Desbrow et al., 2014; Seery and Jakeman, 2016; Costa et al., 2020a). Collectively, these results demonstrate that some mild discomfort associated with EIGS may be expected following consumption of both sucrose alone, and sucrose-lactose-protein containing beverages, even for individuals without lactose intolerance or dairy protein allergies. Findings from the current study suggest that individual tolerance (i.e., total volume, nutrient concentration, and intake dosage timing) and intestinal absorptive capacity are more likely determinants of carbohydrate malabsorption, than the broad recovery beverage composition *per se*. Nevertheless, based on the current findings, a pragmatic approach to professional practice would be to initiate recovery nutritional provisions immediately after exercise cessation, in small and frequent doses over the 1–2 h acute recovery time period, as to avoid gastric and intestinal nutrient overload and allow gastrointestinal patency to return as a result of EIGS; noting that habitual dietary patterns should return to normal 2 h into recovery.

### Glucose Availability to Recovery Nutrition

Glucose availability appears to be largely influenced by individual tolerance and metabolic status; whereby, appetite, GIS, intake content (i.e., nutrient, volume, and timing), and additional fuel requirements in the post-exercise period (e.g., excess post-exercise O_2_ consumption for metabolic re-establishment, tissues repair, immune function, thermoregulation, then surplus for glycogen resynthesis) may influence the overall fuel replacement outcomes. Despite the lower carbohydrate content, CEB resulted in a greater peak and AUC for blood glucose concentration over the 2 h period after beverage ingestion. It was unfortunate that the insulin response for CEB was not detected due to the sampling time points (i.e., 0 and 2 h after beverage ingestion). However, the absent insulin response at 2 h suggests insulin peaked and returned to baseline between the sample time points. Previous studies have reported peak serum insulin values to occur 15 to 30 min following consumption of 0.8 g/kg BM of glucose or sucrose, before returning to basal values at 2 h post-consumption (Bowtell et al., 1999, 2000). Whereas, the greater carbohydrate content of CM resulted in a lower blood glucose peak and AUC, likely due to the greater energy content and protein inclusion, known to slow gastrointestinal transit and intestinal absorption activity (Leiper, 2015). It is possible that the greater overall energy content in addition to the slowed delivery into circulation resulted in a more sustained insulin response, aligned with previous investigations (Williams et al., 2003; Lunn et al., 2012). Indeed, protein co-ingestion with carbohydrate has shown to induce a synergistic insulin response, greater than equivalent or lower energy intake of carbohydrate alone (Zawadzki et al., 1992; Van Loon et al., 2000). These two distinct glucose availability trajectories suggest the gastrointestinal tract is a gateway regulatory factor for circulatory nutrient provisions to support the other elements of exercise recovery.

### Immune Responses to Recovery Nutrition

A single bout of prolonged strenuous exercise is proposed to promote acute innate immune altering properties (Walsh et al., 2011b; Suzuki, 2018). Restoration of immunocompetency seems necessary to prevent environmental and luminal derived pathogenic agents overriding the body’s defenses leading to increased risk of infection and (or) delayed recovery (Walsh et al., 2011a; Costa et al., 2017a; Peake et al., 2017). In the current study, an exercise-induced leukocytosis, neutrophilia, and increased neutrophil:lymphocyte ratio was observed. The type of recovery beverage consumed did not influence leukocyte trafficking or prevent a decrease in neutrophil degranulation (CM: −38% vs. CEB: −56%). We have previously demonstrated that consumption of a recovery beverage of similar carbohydrate-protein composition, immediately after prolonged strenuous exercise (e.g., 2 h running at ≥70% *V̇*O_2__max_ in temperate ambient conditions) prevented the exercise-associated reduction in *in vitro* bacterially challenged neutrophil functional responses (Costa et al., 2009, 2011, 2020a). It is postulated that the 1 h delay in providing the recovery beverage in the current study abolished any immune-enhancing effects of the dairy milk beverage, as previously demonstrated with delayed versus immediate feeding, and provisions of water only (Costa et al., 2009, 2011, 2020a). Given that phagocytic immune function is fundamental for clearance of luminal-derived pathogenic agents, and muscle tissue de- and regeneration (Suzuki, 2018), enhanced immune function associated with immediate provision of a nutritive recovery beverage may be supportive of broader aspects of acute recovery optimization.

In the current study, there was no substantial systemic inflammatory cytokine response, except for the modest post-exercise increase in anti-inflammatory cytokines IL-10 and IL-1ra. These outcomes suggests, 2 h HIIT creates no to minimal consequence to systemic inflammatory status, and that anti-inflammatory cytokine markers (e.g., IL-10 and IL-1ra) appear to be more sensitive to exercise stress. Findings are consistent with previous research using similar exercise stress (Suzuki et al., 2020), and same experimental controlled procedures and conditions (Gill et al., 2015a, b, 2016; Snipe et al., 2018a, b; Costa et al., 2019; Gaskell et al., 2020a, b), whereby systemic inflammatory cytokine responses are characteristic of none to small increases in pro-inflammatory cytokine markers (i.e., TNF-α and IL-1β), none to modest increases in systemic response cytokine markers (i.e., IL-6 and IL-8), and modest to large increases in anti-inflammatory cytokine markers (i.e., IL-10 and IL-1ra). The translocation of bacterial endotoxins from the lumen into circulation is reported as a prime contributing factor to the systemic inflammatory response peaking during the exercise recovery period (Peake et al., 2015; Costa et al., 2017b). Considering there were no substantial increases in plasma sCD14 or LBP concentrations (i.e., indirect markers for luminal translocated bacterial endotoxin), it is not surprising that systemic inflammatory responses were minimal. In addition, inflammatory cytokines were unaltered by the recovery beverage intervention, as previous reports suggest that certain foods have the propensity to acutely alter systemic cytokine profile in the post-prandial period (Cowan et al., 2020).

### Muscle Glycogen and Recovery Nutrition

The availability of stored skeletal muscle glycogen is a major determinant of prolonged exercise performance. A major finding of the current study was that consumption of both CM, with a nutritional composition equivalent to current recovery nutrition guidelines (Thomas et al., 2016), and CEB (suboptimal carbohydrate and non-nitrogenous) failed to achieve a significant increase in muscle glycogen restoration in the acute recovery period (i.e., 2 h post-exercise, 90 min after consumption). This occurred despite the exercise protocol employed was in accordance with previous exercise-induced muscle glycogen depleting protocols (Areta and Hopkins, 2018), and post-exercise circulatory glucose and insulin responses that suggest delivery of carbohydrates into circulation and subsequent uptake into insulin-sensitive tissues. Likewise, these responses suggest that nutrient availability of both beverages was not limited by absorption at the level of the gastrointestinal tract. A heightened insulin response and associated phosphorylation of GSK3-β with carbohydrate-protein provisions on CM suggests enhanced cellular activity toward glycogen disposal. Our failure to detect acute changes in muscle glycogen concentrations as a result of recovery beverage consumption could likely be attributed to: (1) reduced glycogen storage capacity due to compromised skeletal muscle cell structural integrity, (2) reduced translocation of the GLUT-4 transporter to the plasma membrane, and (or) (3) reduced insulin sensitivity and (or) GLUT-4 effectiveness associated with damaged skeletal muscle plasma membrane sustained during eccentric plyometric muscle contractions (Asp et al., 1995). The current study did not collect samples to measure muscle damage biomarkers (e.g., CK or myoglobin) beyond the 4 h recovery post-exercise period, or to measure muscle glycogen restoration over the full recovery period. Therefore, a potential limitation within the current protocol is the inability to quantify muscle glycogen prior to or 24 h after exercise, and the extent to which muscle damage may have contributed to the impaired glycogen restoration or the duration of the effect. Additionally, in hindsight, the current study could have integrated a practical measurement test on the 2nd day of testing, such as vertical jump on a force platform (Huschtscha et al., 2020), to assess the degree of exercise-induced muscle damage and implications on performance. It is also worth noting that the authors acknowledge the ethical issues, participant burden, and potential confounding factor of performing running HIIT with eccentric plyometric contractions and running performance test after a muscle biopsy sample. The effect of plyometric exercise induced muscle damage on muscle glycogen resynthesis, including the ideal nutrient intake and time course for repletion, warrants further investigation, given that current guidelines regarding dose and timing of carbohydrate intake may not be applicable to sports that involve HIIT interspersed with eccentric exercise (e.g., team sports and combat sports).

### Muscle Protein and Recovery Nutrition

The progression of skeletal muscle repair and adaptation following prolonged strenuous exercise, and nutrition support, has only recently begun to be investigated (Moore et al., 2014). Consistent with previous literature, we found that provision of 0.4 g/kg BM of protein in the CM beverage induced a greater insulinemic effect and enhanced upregulation of protein synthetic pathways, as indicated by greater phosphorylation of mTOR compared to the non-nitrogenous CEB (Moore et al., 2014). The superior effect of CM on insulin responses and skeletal muscle protein synthesis signaling pathways is likely due to the protein quality, specifically the leucine content, and overall greater carbohydrate content (Phillips et al., 2005). Indeed, phosphorylation of the Akt-mTOR-rpS6 signaling pathway is preceded by nutrient- and contraction-induced activation of the insulin-IGF signaling pathway. Increased Akt-mTOR-S6K phosphorylation has been observed after resistance, endurance and concurrent exercise protocols (Camera et al., 2010), with further enhancement associated with post-exercise protein intake (Breen et al., 2011; Camera et al., 2015; Rowlands et al., 2015). We acknowledge that changes in this signaling pathway have not been consistently correlated to changes in fractional synthesis rate and long-term functional outcomes. However, due to the current experimental procedures (e.g., exercise protocol, sample timing, type and amount of blood and tissue sampling), it was not experimentally feasible to perform continuous isotopic tracer infusions, thus posing a limitation in quantification of muscle protein synthesis. Indeed, since kinetic measures of skeletal muscle protein synthesis and intracellular activity in response to exercise and nutritive interventions are limited to highly invasive, expensive, and complex techniques, researchers face restricted feasibility with regards to exercise and experimental protocols. Nonetheless, the current study provides further evidence of superior effects on anabolic signaling following consumption of the carbohydrate-protein CM beverage, compared to the non-nitrogenous CEB. A noted limitation within the current study is the low rates of consent to the muscle biopsy procedure by female athletes, possibly due to the high burden and invasive nature of the procedure. Research investigating post-exercise nutritional strategies to maximize MPS is an emerging area of research, and to date has exclusively employed male athletes. Characterization of sex-based differences in post-endurance exercise MPS and nutrient requirements warrants further investigation (Russo et al., 2020).

### Hydration and Recovery Nutrition

Exercising in a hypohydrated state has been shown to increase physiological strain, and has potential to decrease physical and mental performance (Costa et al., 2014, 2019; McCartney et al., 2018). Despite the prolonged and strenuous nature of the exercise protocol conducted in thermoneutral conditions with minimal water intake, a substantial degree of hypohydration was not observed on either trial. *P*_Osmol_ and TBW remained within range of euhydration throughout both experimental trials, and were similar to those previously reported after 2 h continuous running at 75% *V̇*O_2__max_ (Costa et al., 2009, 2011). As such, these conditions are not conducive to assessing the overall efficacy of a rehydration beverage. Indeed, blood (i.e., *P*_Osmol_, plasma electrolyte concentrations, plasma aldosterone), body water (i.e., BM change, TBW), and fluid intake tolerance (i.e., rating of thirst and fluid volume) markers of hydration applied within the current protocol failed to detect significant differences in fluid dynamics associated with each recovery beverage. However, significant differences were detected for urine output, fluid retention and Δ*P*_V_, suggestive of greater osmotic potential exerted by the nutrient density of the CM beverage, beyond the greater sodium content of the CEB. It is theorized that the greater nutrient density on CM: (1) promoted a slowed gastrointestinal transit, (2) a more prolonged and distributed intestinal water absorption, (3) prevented acute circulatory hypervolemia and subsequent diuresis, and (or) (4) supported circulatory water retention capacity considering no differences in plasma aldosterone concentration were observed. This theory is supported by the observed differences in blood glucose and insulin responses, indicating more rapid absorption of CEB, despite lower carbohydrate content and comparable malabsorption. Indeed, previous studies examining the hydration potential of carbohydrate-protein beverages after <2% BML have reported enhanced fluid retention and (or) more positive fluid balance when intake of the recovery beverage is isovolumetric and no other food or fluid is consumed (Shirreffs et al., 2007; Desbrow et al., 2014; Seery and Jakeman, 2016). However, when intake is *ad libitum* and participants have access to food and water, this effect is no longer observed (Baguley et al., 2016; Campagnolo et al., 2017). These findings are of practical relevance to athletes, such that aggressive hydration strategies with nutritive beverages will have little to no influence on hydration markers when there is sufficient recovery time and access to meals and water (Sawka et al., 2007). Moreover, researchers investigating hydration and fluid dynamics amongst athletes are encouraged to use multiple, validated methods, including blood, body water and feeding tolerance markers for comprehensive global assessment of intake, gastrointestinal transit, and systemic availability, retention and losses.

### Practical Translation

Many athletes purchase and consume commercial recovery beverages to support recovery outcomes. Flavored dairy milk beverages, in particular, have gained popularity within the sport and exercise community due to the close alignment with current recovery nutrition guidelines. Anecdotally, however, some resistance persists due to the belief that dairy products will induce greater gastrointestinal discomfort. Findings from the present study dispel this notion. Athletes are advised to consume small and frequent doses of nutritional composition equivalent to 1.2 g/kg BM carbohydrate and 0.4 g/kg BM protein over the 1–2 h acute recovery time period, and return to habitual dietary patterns thereafter. It is noted that the nutritional composition of the recovery beverage is inconsequential within the context of overall nutritional intake over a 24 h window, and does not influence subsequent performance 24 h after the initial exercise bout. However, the acute clinical (i.e., minimizing gastrointestinal burden, stimulating neutrophil function toward clearance of tissue debris, endotoxins and luminal-derived pathogenic agents, reduced risk of illness and soft tissue injury) and physiological (i.e., maximized nutrient absorption and hydration, substrate provision for repletion of skeletal muscle glycogen, and substrate and anabolic stimulus for skeletal muscle repair and adaptation) implications of immediate consumption of small and frequent boluses of a dairy milk beverage is likely to provide a cumulative advantage within an extended training regime. Finally, despite the current endurance athlete cohort, the exercise protocol applied within the present experimental design highlights practical implications for athletes participating in sport and exercise activities that involve HIIT interspersed with eccentric and (or) explosive efforts (i.e., may include football, soccer, basketball, and tennis). The effect of plyometric and eccentric activity on muscle glycogen resynthesis, including the ideal nutrient intake and time course for repletion, warrants further investigation.

## Conclusion

The current 2 h HIIT exercise protocol resulted in physiological disturbance (e.g., increased heart rate, RPE, rectal temperature, cortisol, and aldosterone), intestinal injury, GIS, leukocytosis, reduced bacterially-stimulated neutrophil function, modest systemic cytokinaemia (driven by anti-inflammatory cytokines IL-10 and IL-1ra), muscle glycogen depletion, and modest fluid losses without disturbances to plasma electrolyte status. Carbohydrate malabsorption and GIS was evident during the recovery period on both CM and CEB. A more rapid rate of glucose availability and corresponding lower acute insulin response was observed on CEB, compared with CM. Post-exercise leukocyte trafficking, depressed neutrophil function in response to bacterial challenge, and modest systemic inflammatory responses were not affected by the different recovery beverage composition. A greater fold increase in p-GSK-3β/total-GSK-3β was observed on CM compared with CEB; however, neither beverage achieved net muscle glycogen re-storage in early recovery. The protein-containing CM resulted in greater phosphorylation of mTOR, but no effects of beverage consumption occurred for Akt or rpS6. Despite lower sodium content, greater fluid retention was observed following consumption of the carbohydrate-protein beverage (CM) compared to carbohydrate only (CEB); indicative of a lower acute *P*_V_ hypervolemia, and subsequently lower urine production and output. Physiological and performance outcomes the following day did not differ between trials. Recovery optimization proposes that recovery aspects are not isolated systems, but rather interconnected; and appear to respond differently, albeit subtly and with individual variation, to the nutrient composition of recovery nutrition. Nevertheless, these findings suggest that small, frequent doses of a flavored dairy milk beverage is well-tolerated supports greater overall recovery optimization during the acute recovery period (i.e., 2 h post-exercise) compared to a non-nitrogenous carbohydrate-electrolyte beverage.

## Data Availability Statement

The raw data supporting the conclusions of this article will be made available by the authors, without undue reservation.

## Ethics Statement

The studies involving human participants were reviewed and approved by Monash University Human Research Ethics Committee. The patients/participants provided their written informed consent to participate in this study.

## Author Contributions

RC was the chief investigator of this research. IR, JP, and LB contributed toward development of the experimental design. All other authors contributed toward various aspects of data collection and sample collection and analysis. IR and RC contributed to the analysis of the raw data. IR and RC prepared the original draft manuscript. All authors contributed to the review and final preparation of the manuscript.

## Conflict of Interest

The authors declare that the research was conducted in the absence of any commercial or financial relationships that could be construed as a potential conflict of interest.
